# Supplementary figures and images for: Tafazzin regulates neutrophil maturation and inflammatory response
Source: EMBO Rep. 2025 Feb 17;26(6):1590–619. doi: 10.1038/s44319-025-00393-w (PMC11933368; doi:10.1038/s44319-025-00393-w)

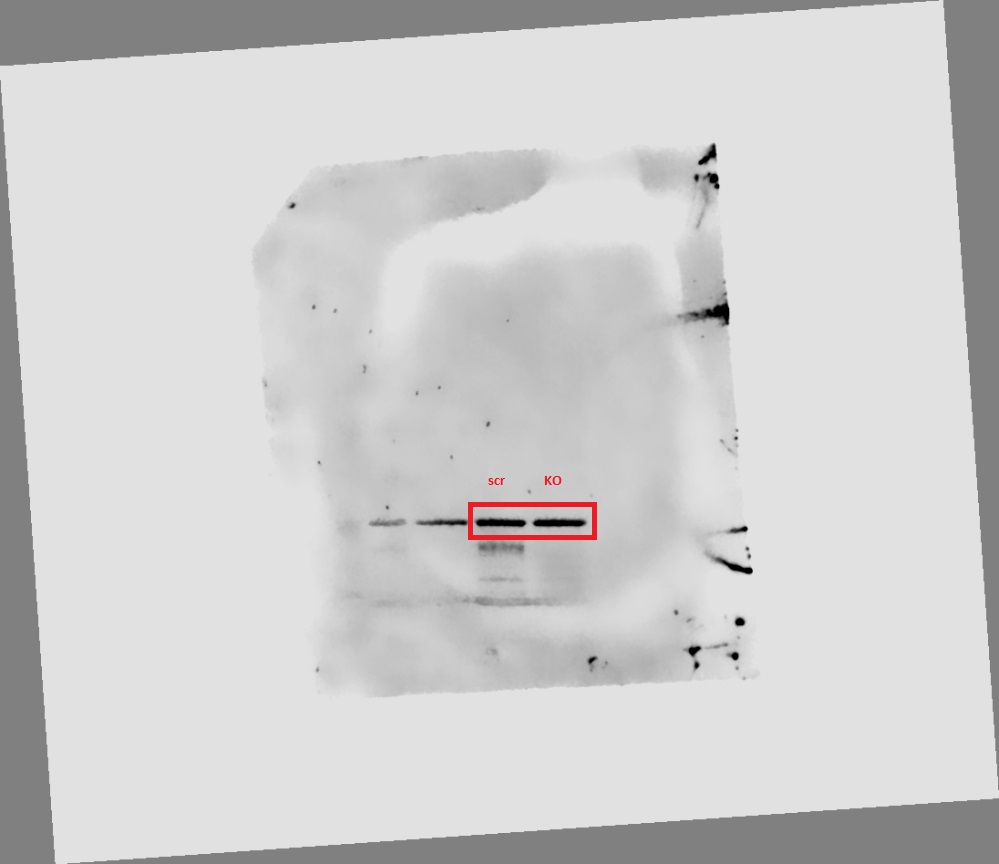

Supplement: Supplementary file 3 — Source data Fig. 2 [file 44319_2025_393_MOESM3_ESM.zip › Figure 2/2C/GAPDH WB.jpg]

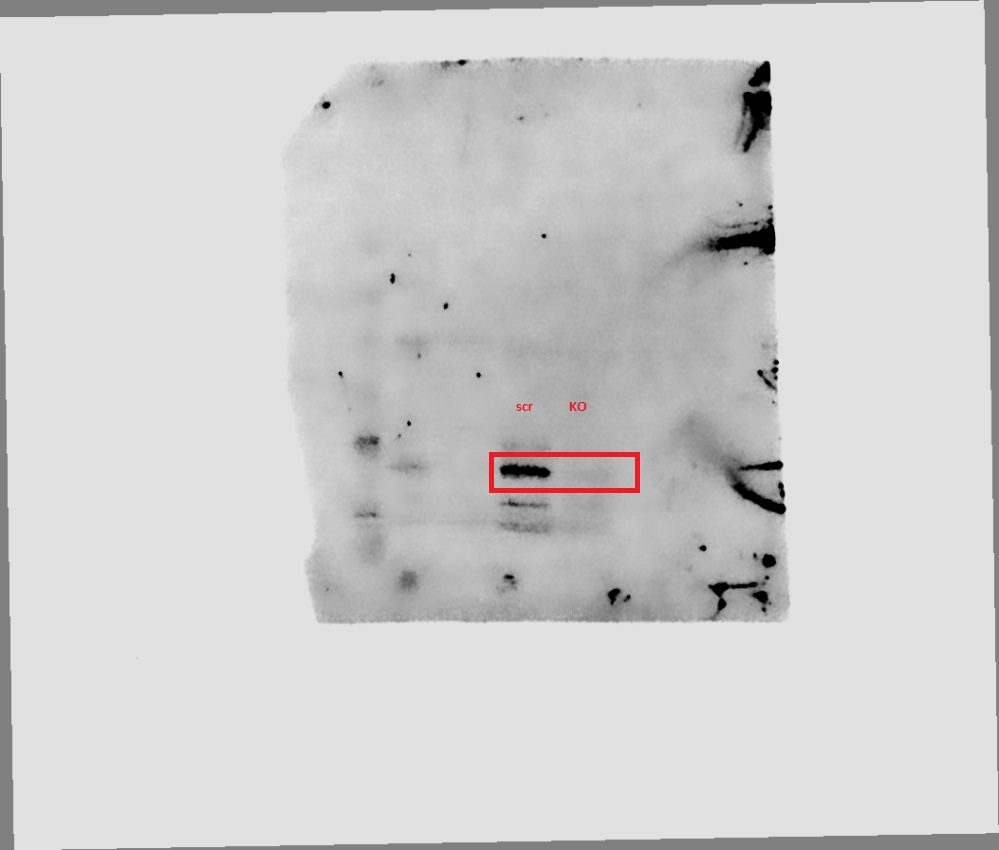

Supplement: Supplementary file 3 — Source data Fig. 2 [file 44319_2025_393_MOESM3_ESM.zip › Figure 2/2C/tafazzin WB.jpg]

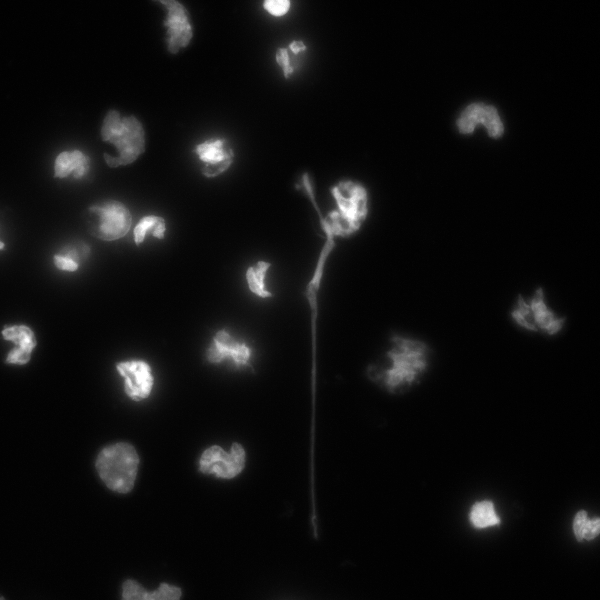

Supplement: Supplementary file 6 — Source data Fig. 5 [file 44319_2025_393_MOESM6_ESM.zip › Figure 5/5G/control CRISPR A23-stimulated NETs.tif]

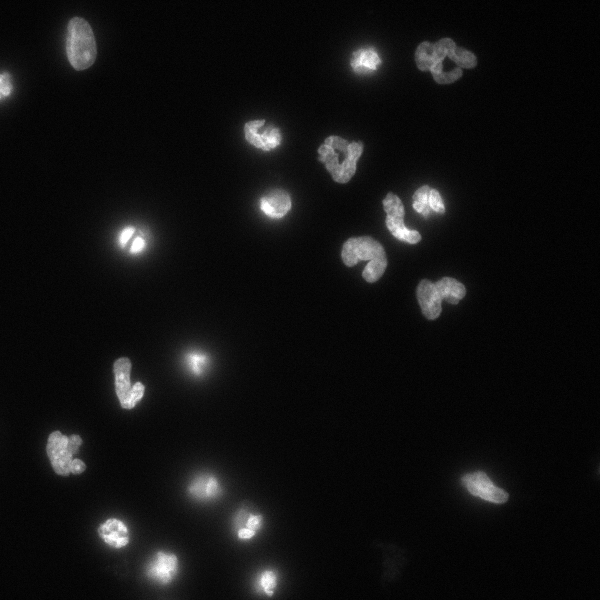

Supplement: Supplementary file 6 — Source data Fig. 5 [file 44319_2025_393_MOESM6_ESM.zip › Figure 5/5G/control CRISPR unstimulated NETs.tif]

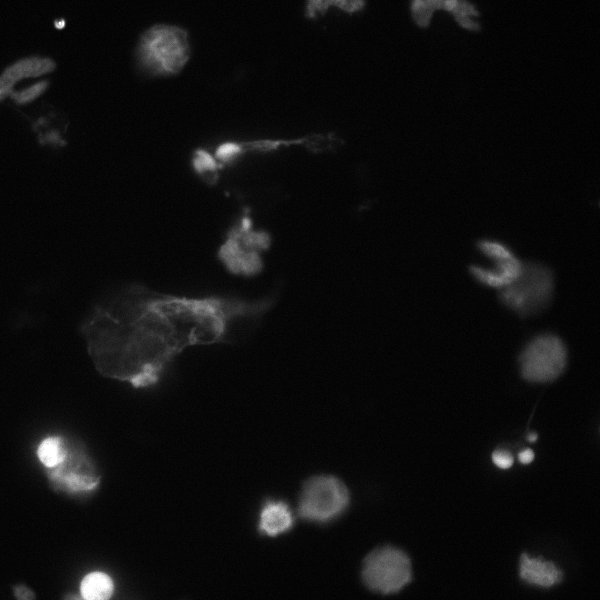

Supplement: Supplementary file 6 — Source data Fig. 5 [file 44319_2025_393_MOESM6_ESM.zip › Figure 5/5G/TAFAZZIN CRISPR A23-stimulated NETs.tif]

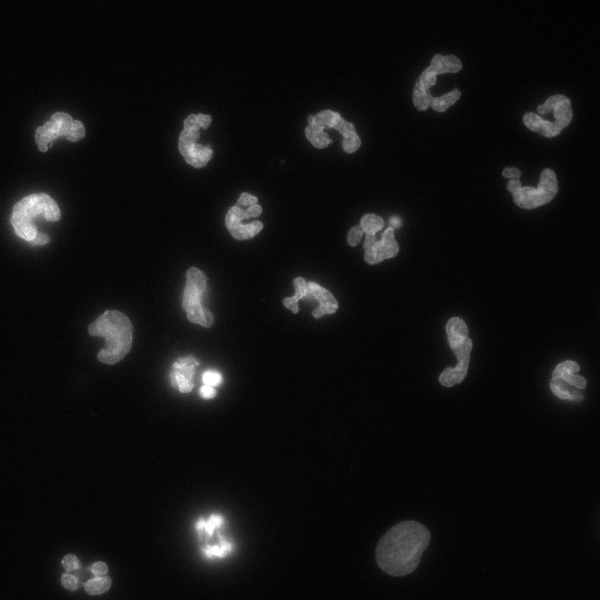

Supplement: Supplementary file 6 — Source data Fig. 5 [file 44319_2025_393_MOESM6_ESM.zip › Figure 5/5G/TAFAZZIN CRISPR unstimulated NETs.tif]

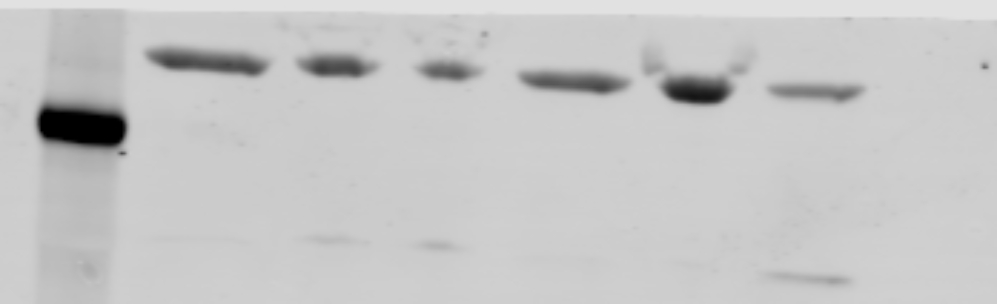

Supplement: Supplementary file 7 — Source data Fig. 6 [file 44319_2025_393_MOESM7_ESM.zip › Figure 6/6D/anti-actin BTHS cultured neutrophils.jpg]

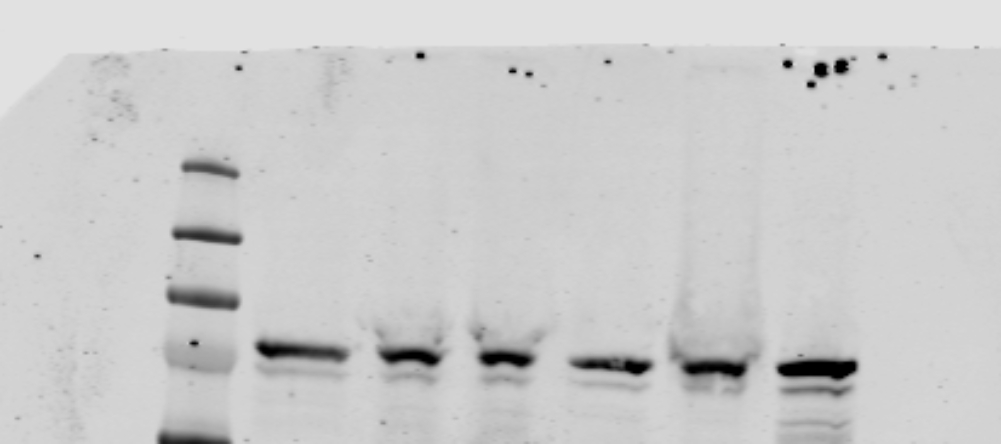

Supplement: Supplementary file 7 — Source data Fig. 6 [file 44319_2025_393_MOESM7_ESM.zip › Figure 6/6D/anti-BiP BTHS cultured neutrophils.jpg]

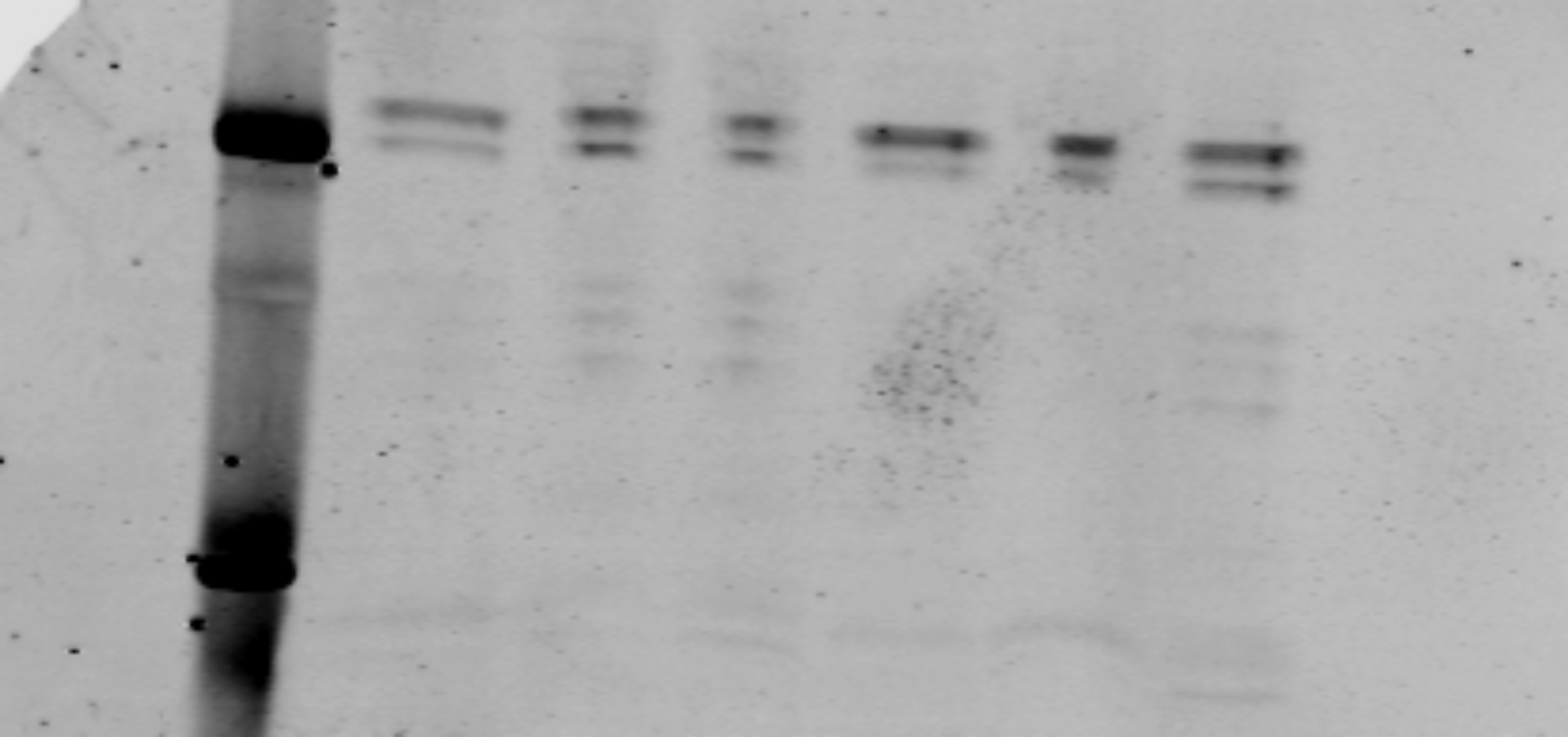

Supplement: Supplementary file 7 — Source data Fig. 6 [file 44319_2025_393_MOESM7_ESM.zip › Figure 6/6D/anti-eIF2a BTHS cultured neutrophils.tif]

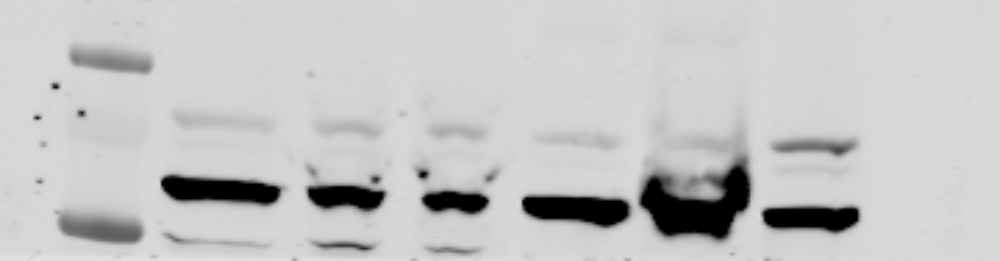

Supplement: Supplementary file 7 — Source data Fig. 6 [file 44319_2025_393_MOESM7_ESM.zip › Figure 6/6D/anti-HSP60 BTHS cultured neutrophils.jpg]

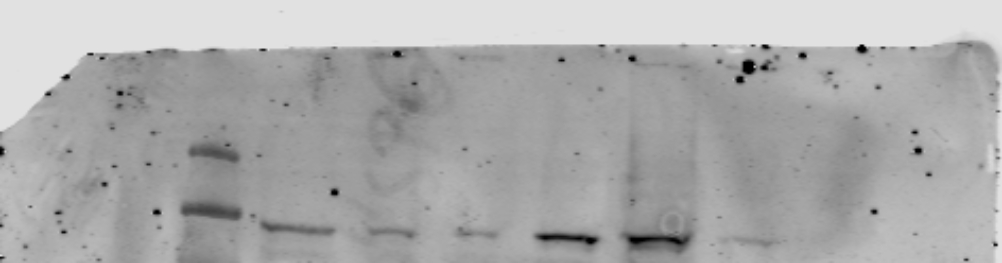

Supplement: Supplementary file 7 — Source data Fig. 6 [file 44319_2025_393_MOESM7_ESM.zip › Figure 6/6D/anti-IRE1a BTHS cultured neutrophils.jpg]

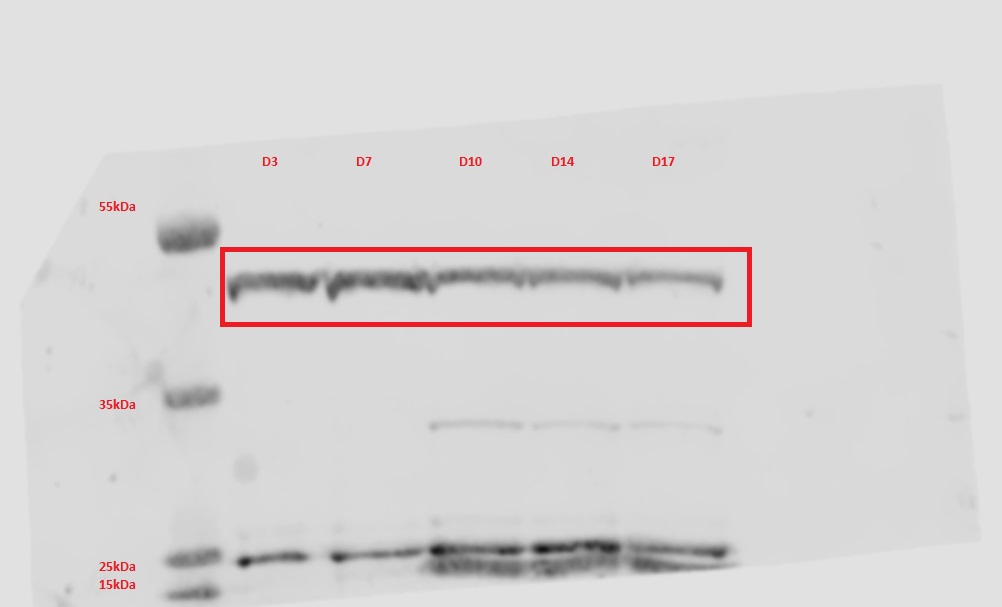

Supplement: Supplementary file 7 — Source data Fig. 6 [file 44319_2025_393_MOESM7_ESM.zip › Figure 6/6E/anti-p-actin n3 061224.jpg]

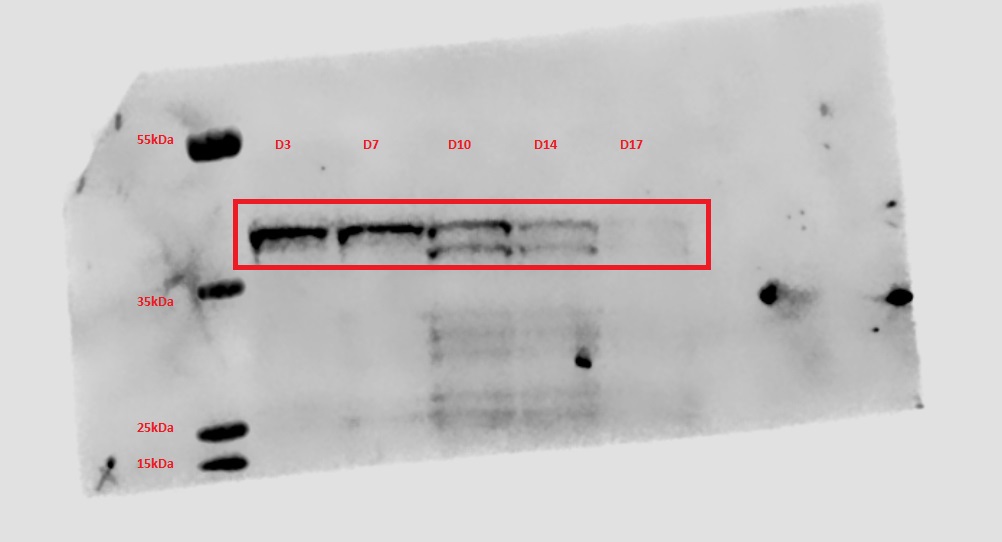

Supplement: Supplementary file 7 — Source data Fig. 6 [file 44319_2025_393_MOESM7_ESM.zip › Figure 6/6E/anti-p-eIF2a n3 061224.jpg]

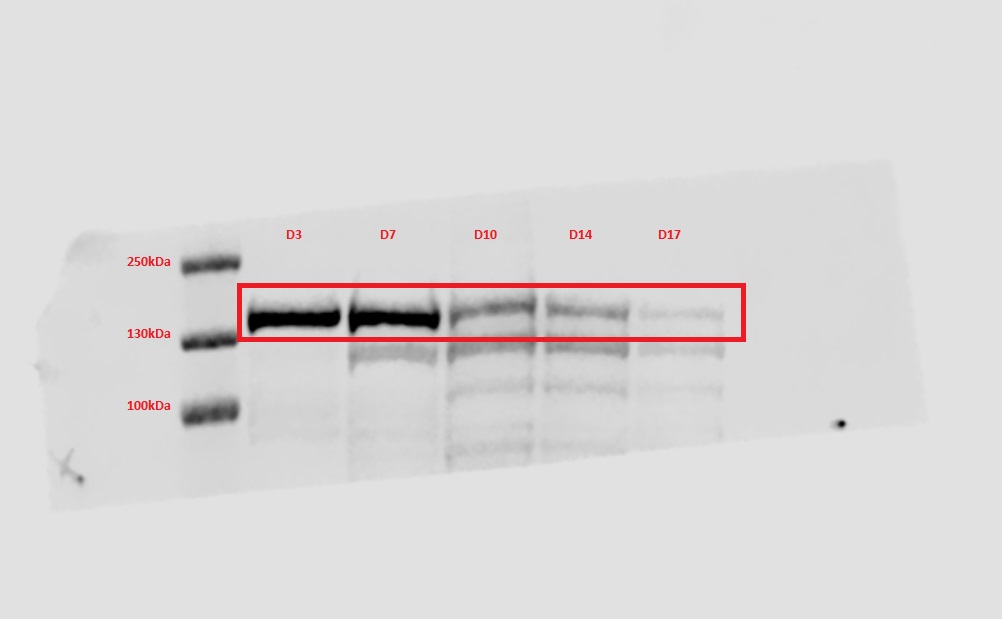

Supplement: Supplementary file 7 — Source data Fig. 6 [file 44319_2025_393_MOESM7_ESM.zip › Figure 6/6E/anti-PERK n3 061224.jpg]
