# Supplementary material for: Tafazzin regulates neutrophil maturation and inflammatory response
Source: EMBO Rep. 2025 Feb 17;26(6):1590–619. doi: 10.1038/s44319-025-00393-w (PMC11933368; doi:10.1038/s44319-025-00393-w)
Supplement: Supplementary file 8 — Expanded View Figures [file 44319_2025_393_MOESM8_ESM.pdf]

Expanded View Figures

Figure EV1. Additional analyses of BTHS neutrophils cultured ex vivo.

(**A**) Representative image of BTHS patient bone marrow aspirate stained with Wright-Giemsa. (**B**) Quantification of average percentage of apoptotic (Annexin V + PI-) HSPC-derived neutrophils at the end of differentiation, $n = 2$ (HC), 3 (BTHS). (**C**) Representative cytospins of HSPC-derived neutrophils (day 17). (**D**) Simplified differential count of cytospins of HSPC-derived neutrophils (day 17), $n = 7$ (HC), $n = 7$ (BTHS). (**E**) Gating strategy for HSPC-derived neutrophils (day 17). (**F**) Average number of $CD66b^{+}CD15^{+}$ neutrophils in HSPC-derived cells at the of differentiation (day 17) per 1000 of HSPC cells at the start (day 0), $n = 8$ (HC), 9 (BTHS). (**G**). Average percentage of $CD66b^{+}CD15^{+}CD11^{+}$ cells in live population of HSPC-derived neutrophils at day 17, $n = 8$ (HC), 9 (BTHS); $P = 0.0013$. (**H**) Average percentage of $CD66b^{+}CD15^{+}CD16^{+}$ cells in live population of HSPC-derived neutrophils at day 17, $n = 8$ (HC), 9 (BTHS); $P = 0.0029$. Data information: Data are presented as mean ± SD. ns—not significant, $^{**}P \leq 0.01$, assessed by unpaired t test (**B**, **F–H**) and two-way ANOVA (**D**). Scale bar: 40 µm (**A**), 20 µM (**B**).

© The Author(s)

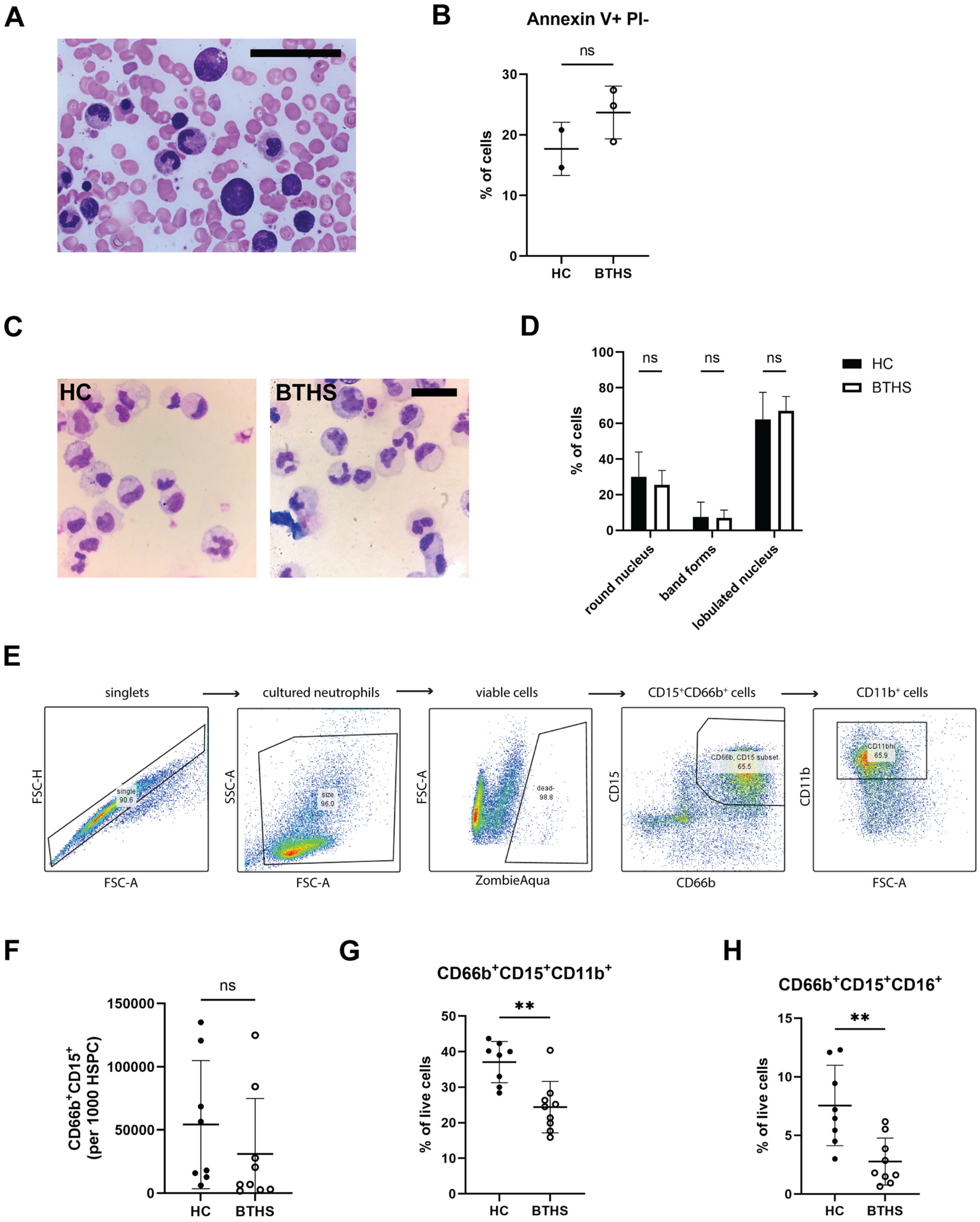
A
B
Annexin V+ PI-
ns
% of cells
HC
BTHS
C
HC
BTHS
D
ns
ns
ns
% of cells
HC
BTHS
round nucleus
band forms
lobulated nucleus
E
singlets
cultured neutrophils
viable cells
CD15⁺CD66b⁺ cells
CD11b⁺ cells
FSC-H
FSC-A
SSC-A
FSC-A
ZombieAqua
CD15
CD66b
CD11b
FSC-A
F
ns
CD66b⁺CD15⁺ (per 1000 HSPC)
HC
BTHS
G
CD66b⁺CD15⁺CD11b⁺
**
% of live cells
HC
BTHS
H
CD66b⁺CD15⁺CD16⁺
**
% of live cells
HC
BTHS

 © The Author(s)

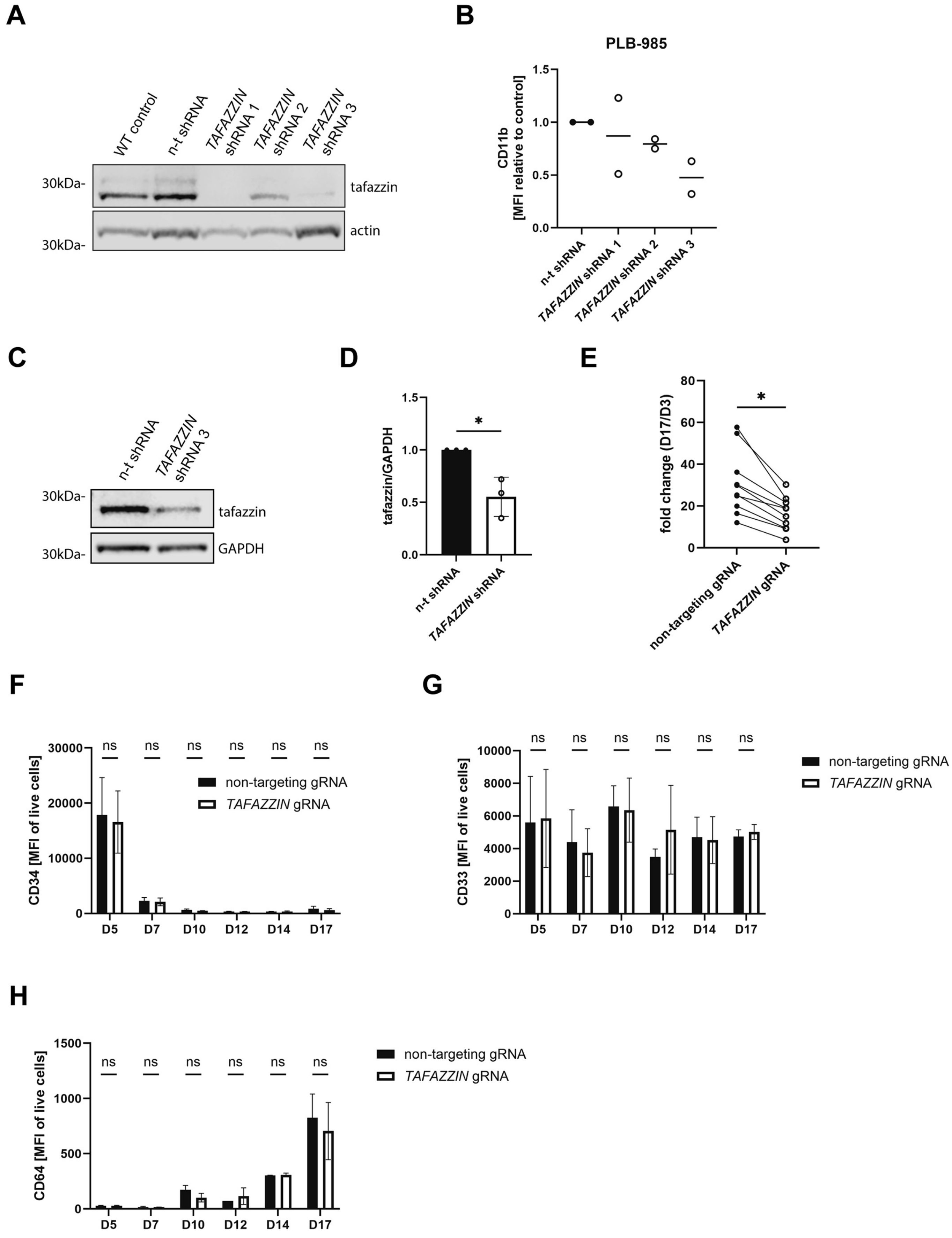
A
WT control
n-t shRNA
TAFAZZIN shRNA 1
TAFAZZIN shRNA 2
TAFAZZIN shRNA 3
30kDa-
tafazzin
actin
30kDa-
B
PLB-985
CD11b [MFI relative to control]
1.5
1.0
0.5
0.0
n-t shRNA
TAFAZZIN shRNA 1
TAFAZZIN shRNA 2
TAFAZZIN shRNA 3
C
n-t shRNA
TAFAZZIN shRNA 3
30kDa-
tafazzin
30kDa-
GAPDH
D
tafazzin/GAPDH
1.5
1.0
0.5
0.0
*
n-t shRNA
TAFAZZIN shRNA
E
fold change (D17/D3)
80
60
40
20
0
*
non-targeting gRNA
TAFAZZIN gRNA
F
CD34 [MFI of live cells]
30000
20000
10000
0
ns ns ns ns ns ns
non-targeting gRNA
TAFAZZIN gRNA
D5 D7 D10 D12 D14 D17
G
CD33 [MFI of live cells]
10000
8000
6000
4000
2000
0
ns ns ns ns ns ns
non-targeting gRNA
TAFAZZIN gRNA
D5 D7 D10 D12 D14 D17
H
CD64 [MFI of live cells]
1500
1000
500
0
ns ns ns ns ns ns
non-targeting gRNA
TAFAZZIN gRNA
D5 D7 D10 D12 D14 D17

Figure EV2. Further characterization of tafazzin-deficient cells.

(**A**) Representative western blot depicting level of tafazzin expression in PLB-985 cells transduced with lentivirus encoding non-targeting (n-t) or anti-*TAFAZZIN* shRNAs, $n = 2$ (experimental repeats). (**B**) Graph showing CD11b surface expression of PLB-985 cells transduced with lentivirus encoding non-targeting (n-t) or anti-*TAFAZZIN* shRNAs, $n = 2$ (experimental repeats). (**C**) Representative western blot depicting level of tafazzin expression level of GFP$^+$ HSPC-derived neutrophils transduced with lentivirus encoding non-targeting (n-t) or anti-*TAFAZZIN* shRNAs, $n = 3$. (**D**) Graph depicting level of tafazzin expression level of GFP$^+$ HSPC-derived neutrophils transduced with lentivirus encoding non-targeting (n-t) or anti-*TAFAZZIN* shRNAs, $n = 3$ (biological repeats); $P = 0.0146$. (**E**) Fold change of total cell count of CRISPR/Cas9-edited HSPC-derived neutrophils from day 3 to day 17 of differentiation matching *TAFAZZIN* knockout cells to their relative controls, $n = 10$ (biological repeats); $P = 0.0154$. (**F**) CD34 surface expression of HSPC-derived neutrophils (live population) during their differentiation in vitro, $n = 3$ (biological repeats). (**G**) CD33 surface expression of HSPC-derived neutrophils (live population) during their differentiation in vitro, $n = 3$ (biological repeats). (**H**) CD34 surface expression of HSPC-derived neutrophils (live population) during their differentiation in vitro, $n = 3$ (biological repeats). Data information: Data are presented as mean ± SD. ns—not significant, $^*P \leq 0.05$, assessed by unpaired *t* test (**D**, **E**) and two-way ANOVA (**F**–**H**).

 © The Author(s)

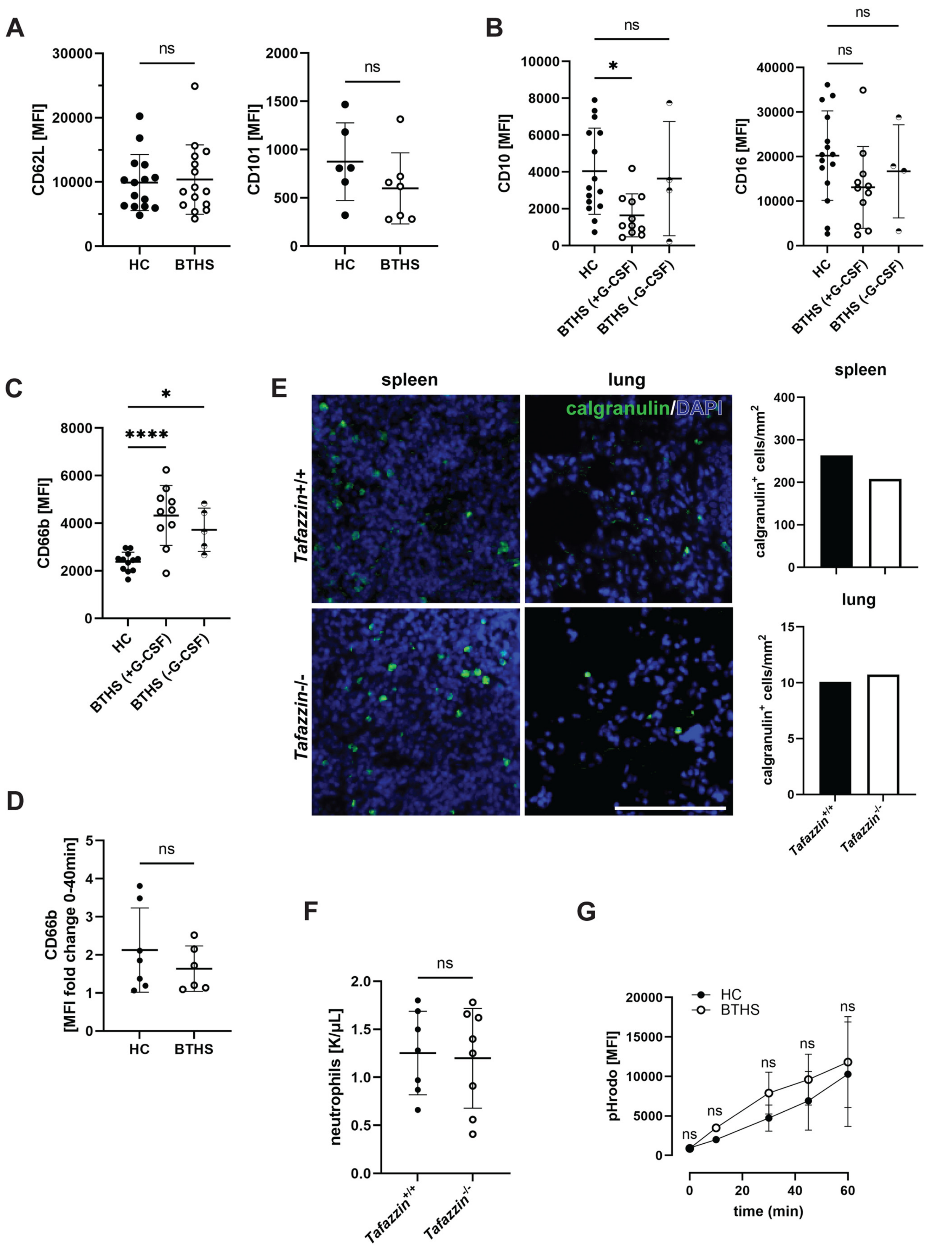
A
CD62L [MFI]
CD101 [MFI]
ns
HC
BTHS
B
CD10 [MFI]
CD16 [MFI]
BTHS (+G-CSF)
BTHS (-G-CSF)
C
CD66b [MFI]
****
*
D
CD66b [MFI fold change 0-40min]
E
spleen
lung
calgranulin/DAPI
Tafazzin+/+
Tafazzin-/-
calgranulin+ cells/mm2
F
neutrophils [K/µL]
G
pHrodo [MFI]
time (min)

Figure EV3. Additional analyses of peripheral blood neutrophils.

(**A**) Surface expression of CD62L (left) and CD101 (right) in circulating neutrophils, $n = 15$ (HC, BTHS). (**B**) Surface expression of CD10 (left) and CD16 (right) in circulating neutrophils stratified according to G-CSF therapy; $n = 15$ (HC), 11 (BTHS + G-CSF), 4 (BTHS − G-CSF). (**C**) Surface expression of CD66b in neutrophils stratified according to G-CSF treatment, $n = 12$ (HC), 10 (BTHS + G-CSF), 5 (BTHS − G-CSF); p (HC vs. BTHS + G-CSF) = < 0.0001, p (HC vs. BTHS-G-CSF) = 0.0187. (**D**) Fold change of CD66b surface expression in isolated neutrophils stimulated with *Streptococcus pyogenes* for 40 min, MOI = 100, $n = 9$ (HC), 7 (BTHS). (**E**) Representative epifluorescence images showing anti-calgranulin (green) and DAPI (blue) staining of mouse lung and spleen (left) and quantification of average number of calgranulin-positive cells per mm^2 of section (right), $n = 2$ (mouse per genotype; the cells were counted from one transverse section through the middle part of the tissue); scale bar = 100 µm. (**F**) Absolute neutrophil counts in mouse whole blood, $n = 7$ (WT), 8 (KO). (**G**) Phagocytosis time-course, measured by pHrodo fluorescence in isolated neutrophils, $n = 3$ (HC, BTHS). Data information: Data are presented as mean ± SD. ns—not significant, *$P \le 0.05$, ****$P \le 0.0001$, assessed by unpaired *t* test (**A**, **D–G**) and one-way ANOVA (**B**, **C**).

© The Author(s)

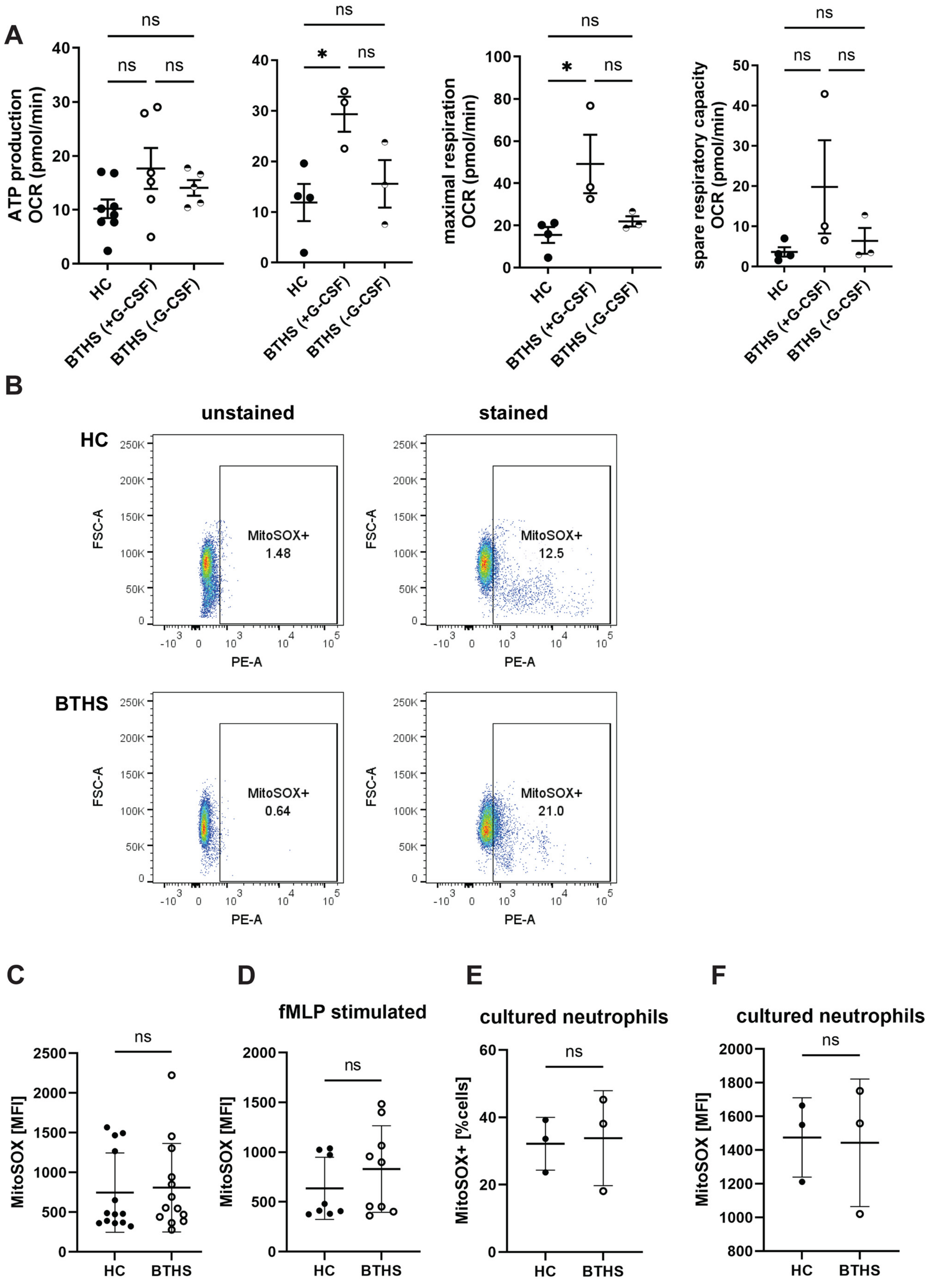
A
ATP production OCR (pmol/min)
maximal respiration OCR (pmol/min)
spare respiratory capacity OCR (pmol/min)
ns
*
HC
BTHS (+G-CSF)
BTHS (-G-CSF)
B
unstained
stained
HC
BTHS
FSC-A
PE-A
MitoSOX+
1.48
12.5
0.64
21.0
C
D
E
F
fMLP stimulated
cultured neutrophils
MitoSOX [MFI]
MitoSOX+ [%cells]
HC
BTHS

Figure EV4. Additional mitochondrial analyses in BTHS neutrophils.

(**A**) Average rates of ATP production, basal respiration, maximal respiration, and spare respiratory capacity, measured by Seahorse metabolic flux analyzer and stratified according to G-CSF therapy; $n = 4$ (HC), 3 (BTHS + G-CSF), 3 (BTHS − G-CSF). (**B**) Gating strategy for MitoSOX+ circulating neutrophils. (**C**) Quantification of average MitoSOX median fluorescence of circulating neutrophils, $n = 13$ (HC, BTHS). (**D**) Quantification of average MitoSOX median fluorescence of circulating neutrophils stimulated with 300 nM fMLP, $n = 8$ (HC), 9 (BTHS). (**E**) Percentage of MitoSOX-positive HSPC-derived neutrophils at the end of differentiation (D17), $n = 3$ (HC, BTHS). (**F**) Percentage of MitoSOX-positive HSPC-derived neutrophils (D17), after stimulation with 300 nM fMLP, $n = 3$ (HC, BTHS). Data information: Data are presented as mean ± SD. ns—not significant, $^*P \le 0.05$, assessed by one-way ANOVA (**A**) and unpaired *t* test (**C**–**F**).

 © The Author(s)

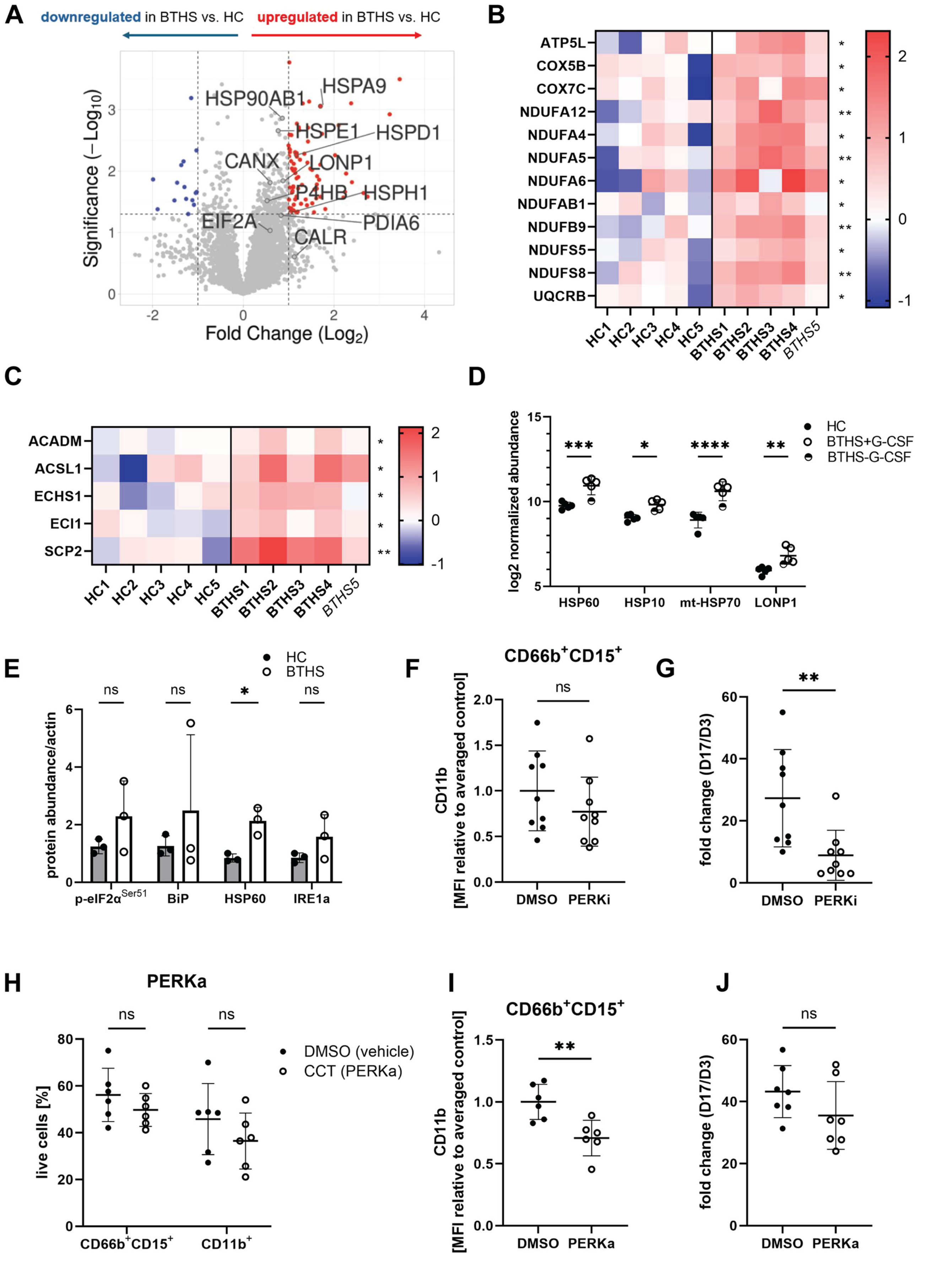
A
downregulated in BTHS vs. HC
upregulated in BTHS vs. HC
HSPA9
HSP90AB1
HSPE1
HSPD1
CANX
LONP1
P4HB
HSPH1
EIF2A
PDIA6
CALR
Significance (−Log10)
Fold Change (Log2)
B
ATP5L
COX5B
COX7C
NDUFA12
NDUFA4
NDUFA5
NDUFA6
NDUFAB1
NDUFB9
NDUFS5
NDUFS8
UQCRB
HC1 HC2 HC3 HC4 HC5 BTHS1 BTHS2 BTHS3 BTHS4 BTHS5
C
ACADM
ACSL1
ECHS1
ECI1
SCP2
D
log2 normalized abundance
HSP60 HSP10 mt-HSP70 LONP1
HC
BTHS+G-CSF
BTHS-G-CSF
E
HC
BTHS
protein abundance/actin
p-eIF2α Ser51
BiP
HSP60
IRE1a
F
CD66b+CD15+
CD11b [MFI relative to averaged control]
DMSO PERKi
G
fold change (D17/D3)
DMSO PERKi
H
PERKa
live cells [%]
DMSO (vehicle)
CCT (PERKa)
CD66b+CD15+
CD11b+
I
CD66b+CD15+
CD11b [MFI relative to averaged control]
DMSO PERKa
J
fold change (D17/D3)
DMSO PERKa

© The Author(s)

Figure EV5. Elevated UPR signaling in BTHS neutrophils.

(**A**) Volcano plot comparing BTHS and HC circulating neutrophil protein abundances, displayed as $-\log_{10}$ *P*-value and $\log_2$ fold change, $n = 5$ (HC), 5 (BTHS including $n = 4$ G-CSF-treated and $n = 1$ untreated, non-neutropenic patient). (**B**) Heat map depicting upregulated oxidative phosphorylation proteins identified with IPA, displayed as log2 fold change over averaged HC. (**C**) Heat map depicting upregulated fatty acid oxidation proteins identified with IPA, displayed as log2 fold change over averaged HC. (**B**, **C**) *BTHS5* - non-neutropenic BTHS patient. (**D**) Log_2 normalized abundance of mtUPR-related proteins identified by proteomics in circulating neutrophils, $n = 5$ (HC), 5 (BTHS including $n = 4$ G-CSF-treated and $n = 1$ untreated, non-neutropenic patient); p (HSP60) = 0.0002, p (HSP10) = 0.0192, *P* (mt-HSP60) = < 0.0001, *P* (LONP1) = 0.0064. (**E**) Quantification of UPR-related protein expression in HSPC-derived neutrophils at D17 of differentiation, $n = 3$ (HC, BTHS); *P* (HPS60) = 0.0106. (**F**). Surface expression of CD11b in PERK-inhibited (GSK2606414; 1 µM) or vehicle control (DMSO-treated) HSPC-derived neutrophils at D17 of differentiation, relative to averaged control (on a same day), $n = 9$ (DMSO, PERKi). (**G**) Fold change in total cell count during HSPC differentiation, from day 3 to day 17, after treatment with PERK inhibitor (GSK2606414; 1 µM) or vehicle control (DMSO), $n = 9$ (DMSO, PERKi); $P = 0.0064$. (**H**) Average percentage of HSPC-derived neutrophils ($CD66b^{+}CD15^{+}$) and mature neutrophils ($CD66b^{+}CD15^{+}CD11b^{+}$) at D17, after treatment with PERK activator (CCT020312; 1 µM, added on day 3, 7, 10, and 14 of culture) or vehicle control (DMSO), $n = 6$ (DMSO, PERKa). (**I**) Surface expression of CD11b in PERK-activated (CCT020312; 1 µM) or vehicle control (DMSO-treated) HSPC-derived neutrophils at D17 of differentiation, relative to averaged control (on a same day), $n = 6$ (DMSO, PERKa); $P = 0.0054$. (**J**) Fold change in total cell count during HSPC differentiation, from day 3 to day 17, after treatment with PERK activator (CCT020312; 1 µM) or vehicle control (DMSO), $n = 7$ (DMSO, PERKa). Data information: Data are presented as mean ± SD. ns—not significant, $^{*}P \le 0.05$, $^{**}P \le 0.01$, $^{***}P \le 0.001$, $^{****}P \le 0.0001$, assessed by Welch's *t* test after $\log_2$ transformation (**B**, **C**), unpaired *t* test (**D–G**, **I**, **J**) and two-way ANOVA (**H**).

 © The Author(s)
